# The Treatment of Goiter

**Published:** 1918-09

**Authors:** Leigh F. Watson

**Affiliations:** 30 N. Michigan Avenue; Chicago


### The Treatment of Goitre.

LEIGH F. WATSON, M. D., CHICAGO.

---

About five years ago I observed the effect of a concentrated solution of quinin and urea which I injected into an angioma of the tongue, that had been repeatedly cauterized with carbon dioxid snow without reducing its size or vascularity. Injections of a fifty per cent. solution caused the tumor to disappear completely; the tongue assumed its normal size and color and there has been no return of the condition since.

Shortly after treating the angioma, I used the quinin and urea injections in a case of severe toxic exophthalmic goiter. The patient was too ill to stand even the slight operation involved in ligating the superior thyroid arteries; the improvement following the injection was more decided than I had ever seen following ligation.

Injections of iodine, carbolic acid, alcohol, arsenic chloroform, iodoform and chromic acid are to be condemned because of their poisonous and corrosive properties and the liability of producing an embolus if accidentally injected into the blood stream. The harmlessness of quinin and urea intravenously is well known; in many localities of the South is it the treatment of choice for certain types of malaria.

### GENERAL OBSERVATIONS.

In the treatment of goiter, as in the treatment of many maladies, the surgeon urges that the patient be recommended for operation early. No doubt the best results do follow the operation when it is performed before disease has reached the more serious secondary stage—just as a smooth and comfortable recovery will often follow the proper medical treatment when administered to beginning cases. It is not my purpose to condemn the operation for goiter. Anyone with experience in the treatment of the disease can not doubt its value as a therapeutic procedure, and in many cases it is the only means from which the patient may derive benefit. It is my opinion, however, that only in exceptional cases should it be the first step taken to effect a cure. The mortality is high, the recurrence is frequent; and until a greater number of patients have been cured by it, and until a longer period has elapsed since it came into use, there will always be a question as to whether the patient operated upon, may not suffer at a later time from too little thyroid function. It has been less than twenty years since the operation of partial thyroidectomy for exophthalmic goiter came into general use.

It is a well established fact that the thyroid begins to shrink and its activity decreases after the thirty-fifth year; and if too much of the gland is removed early in life, it is reasonable to believe that disturbing symptoms of hypothyroidism or myxedema are liable to appear at middle age, with increased susceptibility to infections and disease.

I believe it is possible to destroy a smaller amount of thryoid tissue by quinin and urea injections than is usually removed in the ordinary operation of partial thyroidectomy—the symptomatic relief is the same; statistics show that the recurrence is lessened; and the method is without the danger of the operation. It is a question whether the portion of the gland remaining after lobectomy, is any better able to maintain bodily functions than if a lesser amount is destroyed by injections, cautiously given, so that the greatest amount of gland, compatible with the restoration of health, may be saved.

The studies of Wilson have definitely proved the constant association of exophthalmic goiter with primary parenchymatous hypertrophy and hyperplasia of the thyroid gland. Kendall has isolated from the thyroid of exophthalmic goiter an active principle, thyroxin, which when injected into a normal person increases pulse rate, vigor, metabolism and nervous irritability; a very small amount will produce marked symptoms of hyperthyroidism. He also observes that in exophthalmic goiter the secreting capacity of the gland is greatly increased and the reservoir capacity much decreased.

Bearing in mind these pathological changes which accompany exophthalmic goiter, it is obvious that medical treatment which stops short of destroying a portion of the enlarged and hyperactive gland, will at times fail to afford relief from the active symptoms and will also fail to prevent recurrence when the hypersensitive, although quiescent goiter is subjected to severe strain, such as worry, grief, fright, parturition, climacteric, infectious diseases and operative shock.

### TREATMENT.

The necessity of minimizing the slight pain from any intrathyroid injection, by means of local anesthesia, can not be too strongly emphasized. Preliminary injections into the thyroid gland of a few minims of sterile salt solution, followed by injections of sterile water, are necessary to raise the patient's threshold to stimuli, thereby preventing an acute attack of hyperthyroidism which might otherwise follow the first quinin and urea infiltration. As soon as no hyperthyroidal reaction follows the water injections, their usefulness is at an end.

The strength of the quinin and urea solution varies, depending upon the type of the goiter and the character of the symptoms. Only one injection is given at a treatment, which is repeated at two to six (days) intervals. Ten to twenty infilterations are usually required to produce marked improvement in general symptoms. Too frequently physicians try to hurry the treatment with unpleasant effects on the patient. The first injections of quinine and urea are usually given at the upper pole; when the thrill over the superior thyroid has diminished, the lower pole is injected, and finally the central portion of the gland is treated. It is important that only a few minims of the concentrated quinin and urea solution be given at each treatment if the object of the injection is to cause the disappearance of the goiter without a subsequent operation: massive infiltrations are indicated only when the plan is to follow a course of injections by an immediate partial thyroidectomy.

The toxic cases should be watched carefully, and at the first sign of an acute exacberation of hyperthyroidism, treatment should be stopped, a hypodermic of morphine atrophine and digitalin be given, ice bags applied over the thyroid and heart.

Of course it is realized that all patients must remain in bed during the treatment and for a period following, under careful hygienic and dietetic supervision—the length of time depending on their progress. The treatment has been described in detail in the Journal of the American Medical Association and elsewhere.

EFFECT OF THE INJECTIONS ON THE SYMPTOMS.

| | Relieved | Improved | Not Improved |
|---|---|---|---|
| Exophthalmic | .....85 (average 4 mos.) | 15 | 0 |
| Nonexophthalmic | ..84 (average 2 mos.) | 10 | 6 |

EFFECT OF THE INJECTIONS ON THE GOITER

| | Disappeared | Reduced | Not Reduced |
|---|---|---|---|
| Exophthalmic | .....80 (average 5 mos.) | 15 | 5 |
| Nonexophthalmic | ..75 (average 4 mos.) | 12 | 13 |

THE PREVENTION OF GOITER.

Research workers have found the prevention of simple goiter in animals very easy. Physicians living in goiter districts are strongly urged to induce the parents of young children to carry out prophylactic measures before any goiter appears. I prescribe about 5 to 15 minims of the syrup of ferrous iodine, depending on the child's age, once daily for one week in each month, along with suitable dietetic and hygienic measures. The important point is not to stop the treatment too soon.

30 N. Michigan Avenue.

